# Socioeconomic Disparities and Smoking Habits in Metabolic Syndrome: Evidence from Isfahan Healthy Heart Program

**Published:** 2011-08-01

**Authors:** M Gharipour, R Kelishadi, N Toghianifar, A A Tavassoli, A R Khosravi, F Sajadi, N Sarrafzadegan

**Affiliations:** 1 Department of Clinical Biochemistry, Isfahan University of Medical Sciences, Isfahan, Iran; 2 Department of Pediatric Preventive Cardiology, Isfahan Cardiovascular Research Center, Isfahan Cardiovascular Research Institute, Isfahan University of Medical Sciences, Isfahan, Iran; 3 Isfahan Cardiovascular Research Center, Isfahan Cardiovascular Research Institute, Isfahan University of Medical Sciences, Isfahan, Iran; 4 Medicine Faculty, Isfahan University of Medical Sciences, Isfahan, Iran; 5 Hypertension Research Center, Isfahan University of Medical Sciences, Isfahan, Iran; 6 Department of Nutrition, Isfahan University of Medical Sciences, Isfahan, Iran; 7 Director of Isfahan Cardiovascular Research Center, Isfahan Cardiovascular Research Institute, Isfahan University of Medical Sciences, Isfahan, Iran

**Keywords:** Socioeconomic status, Smoking, Metabolic syndrome, Iran

## Abstract

**Background:**

The metabolic syndrome (Mets) consists of major clustering of cardiovascular disease (CVD) risk factors. This study determines the association of socioeconomic determinants and smoking behavior in a population-based sample of Iranians with Mets.

**Methods:**

This cross-sectional survey comprised 12600 randomly selected men and women aged ≥ 19 years living in three counties in central part of Iran. They participated in the baseline survey of a community-based program for CVD prevention entitled” Isfahan Healthy Heart Program” in 2000-2001. Subjects with Mets were selected based on NCEP- ATPIII criteria. Demographic data, medical history, lifestyle, smoking habits, physical examination, blood pressure, obesity indices and serum lipids were determined.

**Results:**

The mean age of subjects with Mets was significantly higher. The mean age of smokers in both groups was higher than non-smokers but with lower WC and WHR. Marital status, age and residency were not significantly different in smokers with Mets and non-smokers with Mets. Smoking was more common in the middle educational group in the income category of Quartile 1-3. Mets was significantly related to age, sex and education. Middle-aged and elderly smokers were at approximately 4-5 times higher risk among Mets subjects. Low education decreased the risk of Mets by 0.48; similarly in non-smokers, 6-12 years of education decreased the risk of Mets by 0.72.

**Conclusion:**

More educated persons had a better awareness and behavior related to their health and role of smoking. In the lower social strata of the Iranian population, more efforts are needed against smoking habits.

## Introduction

The metabolic syndrome (Mets) consists of major clustering of cardiovascular disease (CVD) risk factors.[1] The main CVD risk factors are obesity, hypertension, diabetes, dyslipidemia and unhealthy lifestyle. [2][3] The combination of these risk factors has been shown to predict type 2 diabetes and CVD.[4] It has been suggested that Mets is associated with demographic and potentially modifiable lifestyle factors5 and is comparatively common in societies undergone epidemiological transition and alterations in lifestyle behaviors, typically caused by economic and technological changes.[5][6] Mets is an important health problem in Iran with a prevalence of approximately 21.9% in the general population.[7] In the past decades, the Iranian population has experienced rapid socioeconomic improvements resulting in lifestyle changes leading to increased prevalence of obesity and associated conditions such as diabetes and dyslipidemia, which are considered to be part of the nutritional transition process.[8] Similar to many other developing countries, the prevalence of smoking has increased in recent years in Iran. Smoking has a positive association with Mets in both sexes.[9] Some studies have showed that low socioeconomic status (SES) may increase the risk of CVD by influencing known behavioral risk factors, such as smoking and unhealthy dietary habits.[10][11] However, there is controversy about the effect of smoking and SES factors on Mets. Some studies have reported that smoking has a protective effect against Mets. Other studies have shown that the relationship between environmental factors such as SES and lifestyle may influence the prevalence of Mets. The purpose of this study was to determine the relationship between smoking as modifiable lifestyle behavior and SES in Iranian individuals with Mets, compared with subjects without Mets.

## Materials and Methods

Isfahan Healthy Heart Program (IHHP) is a crosssectional study which began in 2000 to prevent and control CVD risk factors in the Iranian population. This program was conducted in Central Iran. A stratified multi-stage probability sampling method was used in the baseline survey (2001) and the postintervention in 2007. The final sample included 12,600 subjects older than 19 years who had completed the required health examinations in their nearest health center. The details of the study have been described before.[12] All subjects signed written informed consent after full explanation of the study and the procedures involved. This study was approved by the Ethics Committee of Isfahan Cardiovascular Research Center, a WHO collaborating center. Socioeconomic factors as well as family history of diabetes, hypertension or cardiovascular disease (CVD), lifestyle habits including smoking, dietary patterns and physical activity were recorded. In addition, current use of prescribed medications was recorded. All participants were interviewed at home and subsequently attended a clinic for physical examination including blood pressure. After overnight fasting participants were invited to give blood samples for plasma lipids and glucose. Fasting blood glucose (FBG), 2-hour plasma glucose (2hPG) was also measured after 2 hours an after consumption of 75 g of glucose. Serum lipids, including total cholesterol (TC) and triglyceride (TG), were also measured using the relevant fasting blood sample. All the bloodsampling procedures were performed in the central laboratory of the Isfahan Cardiovascular Research Institute. Three blood pressure readings were obtained. The average of the second and third systolic and diastolic blood pressure readings were used in the analyses.[13] Participants were labeled with Mets according to the NCEP- ATPIII criteria when they had at least 3 or more of the following abnormalities: abdominal obesity: WC≥102 cm in men and ≥88 cm in women and 2 or more of the following 4 components: systolic BP ≥130 mmHg and/or diastolic BP ≥85 mmHg; TG ≥150 mg/dl; HDL-C <40 mg/dl in men and <50 mg/dl in women; fasting blood glucose (FBG) ≥110 mg/dl.[14]

Global dietary index (GDI) was calculated to represent the general dietary behavior.[15] Smoking was defined as smoking at least one cigarette per day at the time of study and otherwise as nonsmoker.[16] Five dimensions of SES were measured including occupation, education, residency, marital status and income. Six categories of occupation were defined as public, private, house-wife, unemployed, student and retired. Education was assessed by the highest achieved level of education and by years spent at school based on the Iranian schooling system: Primary (0-5 years), intermediate and high school (6-12 years), university (≥12 years). City residence was used as proxy measures of urbanization. Subjects were asked to name the place where they were living and were classified according to the location as urban or rural. Income was categorized in quartiles according to poverty line in Iran which was 250 US$ monthly.

A trained team checked recorded data for missing values and entry errors. Missing or unreliable data were rechecked by returning the questionnaires to the main cluster. Statistical analysis was done with the SPSS software (Version 15, Chicago, IL, USA). Descriptive data were presented as means with standard division (SD). Standard T test was used for comparison of means of independent groups and Chi Square was used as appropriate and categorical variables among smokers and nonsmokers between subjects with and without Mets. Logistic regression was conducted to assess the determinants of smoking according to SES variables among subjects with Mets. SES and smoking status were entered in one model to estimate their independent effects. P values of <0.05 were considered to be statistically significant.

## Results

This cross-sectional study was performed on 12,514 individuals, 23.2% of whom met the criteria for Mets. Current smokers made up 8.3% of those with and 17% of those without Mets. Table 1 shows the characteristics of study subjects according to Mets and smoking status. The mean age of subjects with Mets was significantly higher than that of subjects without Mets (p<0.001). The mean ages of smokers in both groups were higher than non-smokers (p<0.001). However, smokers in both groups showed lower WC and WHR (p<0.001). There were non-significant differences in biochemical factors among study groups except for TG and HDL. Table 2 illustrates percentage of SES variables in smokers and non-smokers with or without Mets. Our data showed that marital status, age category and residency were not significantly different in smokers and non-smokers with Mets. Occupation, sex, education and income displayed significant differences in the aforementioned categories. Smoking status was not significantly associated with education in the group without Mets. As shown in Table 2, Mets was significantly related to age (p<0.001 in smokers and non-smokers), sex (p<0.001 in smokers and non-smokers), marital status (p=0.202 in smokers and p<0.001 in non-smokers), education (p=0.010 in smokers and p<0.001 in nonsmokers), occupation (p<0.001 in smokers and nonsmokers) and area of residence (p<0.001 in smokers and non-smokers) among both smokers and nonsmokers. Among non-smokers, Mets is significantly related to age, sex, marital status, education, occupation, income and area of residence (p<0.001). Smoking status did not show any significant association with age and marital status in this group. Smoking status did not show a significant association with place of residence in groups with and without Mets. Smoking was more common in the middle educational group (6-12 years of education) by 12.4%, a ratio approximating that of the middle group in the income category (12.3%) (Quartile 1-3). Odds ratios for Mets according to SES are shown in Table 3. Mets is significantly related to age, sex and education (p<0.001). Based on logistic regression analysis, middle-aged and elderly smokers were at approximately 4-5 times higher risk among Mets subjects (p<0.001). Low education decreased the risk of Mets by 0.48 (p=0.040); similarly in non-smokers, 6-12 years of education decreased the risk of Mets by 0.72 (0.63, 0.82). Our results did not reveal a significant relationship between occupation, marital status and income among smoker subjects with Mets.

**Table 1:** Baseline characteristics of the selected participants according to the metabolic syndrome (MetS) and smoking status a

**Parameter**	**Without Mets**			**With Mets**			**P value (for current smokers between MetS groups**	**P value (for non-smokers between MetS groups) **
	** Current smoker **	** Non-smoker **	** P value **	** Current smoker **	** Non-smoker **	** P value **		
Age (years)	37.57 ± 12.77	36.01 ± 14.27	< 0.001	47.59 ± 13.42	47.56 ± 14.59	0.977	< 0.001	< 0.001
Waist circumference	85.56 ± 10.72	87.46 ± 12.25	< 0.001	101.79 ± 10.84	101.97 ± 11.11	0.813	< 0.001	< 0.001
Hip circumference	96.49 ± 8.98	98.89 ± 10.45	<0.001	105.90 ± 9.73	106.87 ± 9.63	0.134	< 0.001	< 0.001
FBG	79.57 ± 19.82	79.65 ± 22.65	0.892	99.36 ± 42.08	97.19 ± 50.32	0.518	< 0.001	< 0.001
2hhp	89.84 ± 39.60	94.32 ± 36.57	< 0.001	123.04 ± 73.85	128.04 ± 76.18	0.343	< 0.001	< 0.001
T.CHOL	192.27 ± 46.04	190.16 ± 47.31	0.103	220.41 ± 53.16	223.57 ± 50.89	0.358	< 0.001	< 0.001
TG	167.83 ± 102.71	143.88 ± 96.28	< 0.001	274.13 ± 141.56	239.34 ± 115.69	< 0.001	< 0.001	< 0.001
HDL	44.95 ± 10.45	48.43 ± 10.69	< 0.001	39.51 ± 9.17	43.93 ± 9.66	< 0.001	< 0.001	< 0.001
LDL	114.68 ± 38.61	113.64 ± 39.64	0.353	129.52 ± 44.86	133.64 ± 43.88	0.198	< 0.001	< 0.001
SBP	110.93 ± 15.14	111.87 ± 16.28	0.024	128.07 ± 21.21	130.08 ± 22.88	0.188	< 0.001	< 0.001
DBP	72.97 ± 9.71	73.63 ± 10.04	0.015	82.40 ± 11.49	82.66 ± 12.78	0.759	< 0.001	< 0.001
Global Dietary Index	1.13 ± 0.26	1.08 ± 0.26	< 0.001	1.03 ± 0.297	0.98 ± 0.29	0.009	< 0.001	< 0.001

^a^ Fasting blood sugar, 2 hhp: Two hours postprandial, T Chol: Total cholesterol, TG: Triglyceride, LDL: Low density lipoprotein, SBP: Systolic blood pressure, DBP: Diastolic blood pressure

**Table 2:** Socio-economic status according to smoking behavior and the metabolic syndrome.

	**Without Mets (n = 9614)**			**With Mets (n = 2900)**		
	**Current smoker (n = 1637)**	**Non-smoker (n = 7977)**	**P value**	**Current smoker (n = 241)**	**Non-smoker (n = 2659)**	**P value**
Place of Residence						
Urban	16.6	83.4	0.067	8.6	91.4	0.250
Rural	18.1	81.9		7.2	92.8	
Occupation						
Public	22.3	77.7	< 0.001	19.4	80.6	< 0.001
Private	32.5	67.5		26	74	
House wife	1.2	98.8		2.4	97.6	
Not Working student	15.3	84.7		22.7	77.3	
Retired	26	74		15.9	84.1	
Marital Status						
Married	18.1	81.9	< 0.001	8.6	91.4	0.202
Single	13	87		6.7	93.3	
Age category						
19–39 years	16.1	83.9	< 0.001	7.7	92.3	0.147
40–59 years	20	80		9.5	90.5	
≥ 60 years	16.1	83.9		7.2	92.8	
Sex						
Female	1.2	98.8	< 0.001	2.3	97.7	< 0.001
Male	29.4	70.6		26	74	
Education						
0–5 year	17.7	82.3	0.447	6.7	93.3	< 0.001
6–12 year	17.1	82.9		12.4	87.6	
> 12 year	16.1	83.9		9.9	90.1	
Income						
< Quartile 1	16.2	83.8	0.016	6.7	93.2	0.010
Quartile 1–Quartile 3	19.7	80.3		12.3	87.7	
> Quartile 3	17	83		8.5	91.5	

**Table 3:** Adjusted odds ratio (OR) for smoking according to socio-economic variables among individuals with metabolic syndrome (Mets).

**Parameter**	**With Mets versus without Mets**			
	** Non-smoker**		** Smoker**	
	**Odds ratio (95 % CI)**	**P value**	**Odds ratio (95 % CI)**	**P value**
Age category				
19–39 year	R	—	R	—
40–59 year	3.24 (2.88–3.65)	< 0.001	3.93 (2.75–5.60)	< 0.001
≥ 60 year	6.04 (5.14–7.11)	< 0.001	5.07 (3.00–8.55)	< 0.001
Sex				
Female	3.39 (2.60–4.43)	< 0.001	1.70 (0.19–15.67)	0.639
Male	R	—	R	—
Place of Residence				
Urban	1.55 (1.38–1.75)	< 0.001	1.39 (0.96–2.04)	0.081
Rural	R	—	R	—
Occupation				
Public	R	—	R	—
Private	0.88 (0.69–1.11)	0.275	0.89 (0.57–1.37)	0.593
House Wife	1.14 (0 .85–1.52)	0.372	4.43 (0.46–42.96)	0.199
Not Working-student	0.55 (0.39–0 .77)	0.001	0.96 (0.496–1.85)	0.896
Retired	1.34 (0.96–1.87)	0.086	0.88 (0.43–1.81)	0.725
Marital Status				
Married	1.35 (1.16–1.58)	< 0.001	—	0.570
Single	R	—	R	—
Education				
0–5 year	R	—	R	—
6–12 year	0.72 (0.63–0 .82)	< 0.001	1.22 (0.85–1.74)	0.285
> 12 year	0.62 (0.48–0 .79)	< 0.001	0.48 (0.24–0.97)	0.040
Income				
< Quartile 1	R	—	R	—
Quartile 1–Quartile 3	1.14 (1.01–1.29)	0.029	1.16 (0.78–1.72)	0.464
> Quartile 3	1.11 (0.92–1.33)	0.287	1.27 (0.75–2.15)	0.383

## Discussion

Our findings support the hypothesis that social determinants, e.g. occupation, education and income, were related to smoking habits in Iranian individuals with Mets. To the best of our knowledge, no previous study has assessed the role of socioeconomic differences in metabolic risk among smokers. Most previous studies have shown different dimensions of SES are inter-correlated and had complex impact on health issues. The impact of some of the SES dimensions on the Mets components might differ in various communities. [17] In our population, education had the strongest role in this regard; so that low education increased the risk of Mets among smokers. However, we previously reported that higher education was a protective factor against smoking in men, but increasing this likelihood among women.[18] In the current study, the income level had significant associations with smoking. We found significant relationship between quartile 1–-3 or medium level of income and smoking habits in Mets, medium level of income increased the risk of Mets among smokers by 1.14 fold.

The ATTICA study showed an inverse association of education level with clinical and biochemical parameters related to CVD. This association was mainly explained by smoking habits, sedentary lifestyle, obesity, dietary habits, and non-management of risk factors.[19] In our population, it seems that in Mets group, non-smokers had better nutritional habits compared with smokers; hypertriglyceridemia and low HDL were significantly more frequent among smokers than non-smokers.

We previously showed the mean level of serum lipids and anthropometric measures were higher in employed than in unemployed individuals and those with a private job.[18] Smoking was more common among private-employed participants, followed by the public employed and students. We found that retired nonsmokers had an increased risk of Mets by 1.34. Engstrom and co-workers found that the association between risk factors and job existed for major and new CVD risk factors.[20] A study in Finland suggested that employees, especially those in managerial and administrative positions had more favorable risk factor profile than self-employed and industrial workers, and farmers.[21]

Some studies reported the increased risk of Mets in those with lower SES may be due to unfavorable health behaviors as smoking habits, sedentary lifestyle, and dietary habits, as well as increased body mass index and non-compliance to treatment.[21][22] Similarly, the results of a Croatian study showed no significant relationship between different SES dimension, especially education and participating in physical activity, but educated persons participated more in sport activities.[23] We did not collect data on sport activities in this sub-study, but we did not document significant difference in the prevalence of abdominal obesity between the groups studied.

Our results showed that smoking was associated with elevated TG and low HDL. Our findings are in line with the study of Maksimović and colleagues in showing higher TG levels in participants with low educational level than others.[24] Previous studies in developing countries showed that based on social condition, Mets was correlated to lower SES, defined by low household income and lower-grade employment .[25]

The present study has several strengths and weaknesses. We examined a large general population sample that included both subjects with and without Mets and covered a wide age range. The main limitation of our study was its cross-sectional design which makes it difficult to address causal relations. The other limitation is the low accuracy of reporting income.

We found that the three dimensions of SES, i.e. education, occupation and income, were associated with Mets in smokers. Education was the most powerful determinant of Mets among smokers, whereas income was the weakest predictor. It is assumed that more educated persons had a better awareness and behavior related to their health. More educated people were aware about the role of smoking on their health more than low educated persons. We suggest more prevention efforts against smoking in the lower social strata of the Iranian population.

## References

[1] Ford ES, Giles WH, Dietz WH (2002). Prevalence of the metabolic syndrome among US adults: findings from the third National Health and Nutrition Examination Survey. JAMA.

[2] Bayturan O, Tuzcu EM, Lavoie A, Hu T, Wolski K, Schoenhagen P, Kapadia S, Nissen SE, Nicholls SJ (2010). The metabolic syndrome, its component risk factors, and progression of coronary atherosclerosis. Arch Intern Med.

[3] Ford ES (2004). The metabolic syndrome and mortality from cardiovascular disease and all-causes: findings from the National Health and Nutrition Examination Survey II Mortality Study. Atherosclerosis.

[4] Xu H, Song Y, You NC, Zhang ZF, Greenland S, Ford ES, He L, Liu S (2010). Prevalence and clustering of metabolic risk factors for type 2 diabetes among Chinese adults in Shanghai, China. BMC Public Health.

[5] Laaksonen MA, Knekt P, Rissanen H, Härkänen T, Virtala E, Marniemi J, Aromaa A, Heliövaara M, Reunanen A (2010). The relative importance of modifiable potential risk factors of type 2 diabetes: a meta-analysis of two cohorts. Eur J Epidemiol.

[6] Zhu S, St-Onge MP, Heshka S, Heymsfield SB (2004). Lifestyle behaviors associated with lower risk of having the metabolic syndrome. Metabolism.

[7] Gharipour M, Kelishadi R, Baghaie M, Rabiei K (2006). Metabolic syndrome in an Iranian adult population. Eur Heart J.

[8] Azizi F, Ghanbarian A, Momenan AA, Hadaegh F, Mirmiran P, Hedayati M, Mehrabi Y, Zahedi-Asl S (2009). Tehran Lipid and Glucose Study Group. Prevention of noncommunicable disease in a population in nutrition transition: Tehran Lipidand Glucose Study phase II. Trials.

[9] Tavassoli AA, Gharipour M, Khosravi A, Kelishadi R, Siadat ZD, Bahonar A, Sadri GH, Sadeghi M, Rabiei K, Sajjadi F, Zarfeshani S, Eshrati B, Shirani S, Sarrafzadegan N (2010). Gender differences in obesogenic behaviour, socioeconomic and metabolic factors in a population-based sample of Iranians: the IHHP study. J Health Popul Nutr.

[10] Zhu S, St-Onge MP, Heshka S, Heymsfield SB (2004). Lifestyle behaviors associated with lower risk of having the metabolic syndrome.. Metabolism.

[11] Park YW, Zhu S, Palaniappan L, Heshka S, Carnethon MR, Heymsfield SB (2003). The metabolic syndrome: prevalence and associated risk factor findings in the US population from the Third National Health and Nutrition Examination Survey, 1988-1994. Arch Intern Med.

[12] Sarraf-Zadegan N, Sadri G, Malek Afzali H, Baghaei M, Mohammadi Fard N, Shahrokhi S, Tolooie H, Poormoghaddas M, Sadeghi M, Tavassoli A, Rafiei M, Kelishadi R, Rabiei K, Bashardoost N, Boshtam M, Asgary S, Naderi G, Changiz T, Yousefie A (2003). Isfahan Healthy Heart Programme: a comprehensive integrated communitybased programme for cardiovascular disease prevention and control. Design, methodsand initial experience. Acta Cardiol.

[13] Sarrafzadegan N, Baghaei A, Sadri G, Kelishadi R, Malekafzali H, Boshtam M, Amania A, Rabiea K, Moatariana A, Rezaeiashtiania AA, Paradisa G, O’Loughlina J (2006). Isfahan healthy heart program: Evaluation of comprehensive, community-based interventions for non-communicable disease prevention. Prev Control.

[14] Sarrafzadegan N, Kelishadi R, Baghaei A, Hussein Sadri G, Malekafzali H, Mohammadifard N, Rabiei K, Bahonar A, Sadeghi M, O'Laughlin J (2008). Metabolic syndrome: an emerging public health problem in Iranian women: Isfahan Healthy Heart Program. Int J Cardiol.

[15] Mohammadifard N, Kelishadi R, Safavi M, Sarrafzadegan N, Sajadi F, Sadri GH, Maghroon M, Alikhasi H, Heydari S, Sarmadi F (2009). Effect of a community-based intervention on nutritional behaviour in a developing country setting: the Isfahan Healthy Heart Programme. Public Health Nutr.

[16] Gharipour M, Kelishadi R, Sarrafzadegan N, Baghaei A, Yazdani M, Anaraki J, Eshrati B, Tavassoli AA (2008). The association of smoking with components of the metabolic syndrome in non-diabetic patients. Ann Acad Med Singapore.

[17] Hanson RL, Imperatore G, Bennett PH, Knowler WC (2002). Components of the “metabolic syndrome” and incidence of type 2 diabetes. Diabetes.

[18] Bahonar A, Sarrafzadegan N, Kelishadi R, Shirani S, Ramezani MA, Taghdisi MH, Gharipour M (2011). Association of socioeconomic profiles with cardiovascular risk factors in Iran: the Isfahan Healthy Heart Program. Int J Public Health.

[19] Panagiotakos DB, Pitsavos CE, Chrysohoou CA, Skoumas J, Toutouza M, Belegrinos D, Toutouzas PK, Stefanadis C (2004). The association between educational status and risk factors related to cardiovascular disease in healthy individuals: The ATTICA study. Ann Epidemiol.

[20] Engstrom G, Hedblad B, Rosvall M, Janzon L, Lindgarde F (2006). Occupation, marital status, and low-grade inflammation: mutual confounding or independent cardiovascular risk factors?. Arterioscler Thromb Vasc Biol.

[21] Luoto R, Pekkanen J, Uutela A, Tuomilehto J (1994). Cardiovascular risks and socioeconomic status: differences between men and women in Finland. J Epidemiol Community Health.

[22] Chichlowska KL, Rose KM, Diez-Roux AV, Golden SH, McNeill AM, Heiss G (2008). Individual and neighborhood socioeconomic status characteristics and prevalence of metabolic syndrome: the Atherosclerosis Risk in Communities (ARIC) Study. Psychosom Med.

[23] Tahirovic E, Begic H, Sutovic A, Tahirovic H (2010). Impact of the family socioeconomic status on health related quality of life in children operated on for congenital heart defects. Acta Med Croatica.

[24] Maksimovic MZ, Vlajinac HD, Radak DJ, Maksimovic JM, Marinkovic JM, Jorga JB (2008). Association of socioeconomic status measured by education and risk factors for carotid atherosclerosis: cross-sectional study. Croat Med J.

[25] Perel P, Langenberg C, Ferrie J, Moser K, Brunner E, Marmot M (2006). Household wealth and the metabolic syndrome in the Whitehall II study. Diabetes Care.

